# Optical Contact Lenses Biosensors

**DOI:** 10.1021/acssensors.5c01222

**Published:** 2025-10-02

**Authors:** Xiaoye Xia, Yubing Hu, Nan Jiang, Ali K. Yetisen

**Affiliations:** † Department of Chemical Engineering, 4615 Imperial College London, South Kensington, London SW7 2BU, U.K.; ‡ West China School of Basic Medical Sciences & Forensic Medicine, Sichuan University, Chengdu 610041, China; § Jinfeng Laboratory, Chongqing 401329, China

**Keywords:** contact lens sensor, optical biosensing, ocular diseases, neurodegenerative diseases, microfabrication techniques

## Abstract

Tear fluid contains a diverse array of biomarkers reflective of both ocular and systemic health, making it a valuable medium for noninvasive diagnostics. Contact lens biosensors, integrated with optical sensing technologies, provide a promising platform for real-time, continuous monitoring of tear fluid composition. This review focuses on recent advances in the development of contact lens biosensors for optical detection of tear-based biomarkers. Key components include biocompatible lens materials, such as hydrogels and silicone hydrogels, that maintain oxygen permeability and optical clarity, along with fabrication methods such as inkjet printing, micropatterning, and three-dimensional (3D) microfabrication for precise sensor integration. Optical sensing mechanisms, including fluorescence, photonic crystal resonance, and surface plasmon resonance, have demonstrated high sensitivity in detecting glucose, lactate, electrolytes, cortisol, and inflammatory markers at clinically relevant concentrations. Such sensors have shown potential in diagnosing and monitoring diseases including diabetes, dry eye syndrome, stress-related disorders, and neurodegenerative conditions. Despite these advances, challenges remain in minimizing background interference, enabling long-term wear, and achieving multiplexed detection. Future research should prioritize robust biorecognition chemistries, wireless optical readouts, and scalable manufacturing strategies to support clinical translation. Contact lens biosensors are poised to become a key platform in next-generation, personalized healthcare through noninvasive tear fluid analysis.

## Graphical abstract

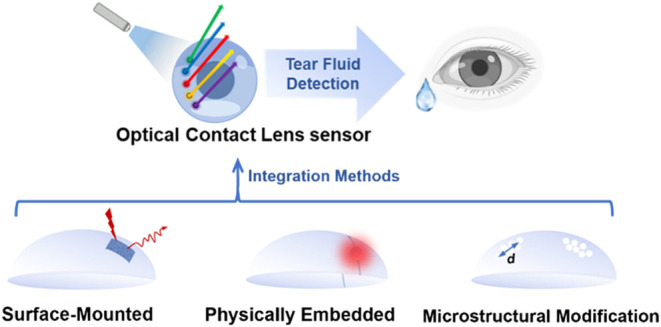

Ocular diseases, including ametropia, glaucoma, and cataracts, significantly diminish patient quality of life and represent a major global health burden. The World Health Organization reported in 2023 that over 2.2 billion people worldwide currently live with visual impairments, at least half of which could be prevented or managed effectively with earlier and more precise diagnostic interventions.^4^ Despite rising demand for personalized healthcare driven by improved living standards, traditional diagnostic tools for ocular diseases remain episodic, invasive, and typically yield only fragmented snapshots of a patient’s condition. Current clinical and point-of-care (POC) diagnostic methods, such as blood tests and ocular examinations, often suffer from limited patient compliance and inconsistent follow-up due to their invasiveness, discomfort, and inconvenience.^6–11^ Thus, there is an urgent clinical need for wearable, continuous, and noninvasive monitoring platforms that deliver real-time, accurate physiological data at the point of care, enabling earlier diagnosis, timely intervention, and truly tailored patient management.

Recent advances in flexible and stretchable substrates have catalyzed the development of conformable, wearable diagnostic platforms capable of continuous, noninvasive health monitoring.^12–15^ In parallel, breakthroughs in microelectronics have facilitated the miniaturization of sensors and onboard signal processing, dramatically reducing the need for bulky instrumentation.^16,17^ Concurrent innovations in optical biosensing—including photonic waveguides, fluorescence-based assays, and plasmonic sensing—now offer unprecedented molecular sensitivity, specificity, and multiplexing capabilities on flexible substrates.^18–21^ Collectively, these technological advancements underpin next-generation wearable biosensors that can continuously detect metabolites, ions, proteins, and physical parameters such as temperature, pressure, and electrophysiological signals directly from biofluids.^22–28^

Among wearable diagnostic devices, contact-lens-based sensors hold distinct advantages, given their direct access to tear fluid and proximity to the corneal surface—both rich sources of biomarkers that closely reflect ocular as well as systemic health conditions, including metabolic disorders, inflammatory diseases, and neurological impairments.^29,30^ Tear fluid is continually replenished at approximately 0.5 μL per minute, contains clinically relevant biomarkers in measurable concentrations, and provides a convenient, painless sampling approach suitable for patient-friendly diagnostics.^33–36^ Additionally, contact lenses are already widely adopted by more than 150 million individuals globally for vision correction and cosmetic purposes, positioning them ideally for seamless integration of sensing functionalities without significant lifestyle disruptions. Indeed, recent pioneering efforts have successfully demonstrated the feasibility of real-time tear glucose monitoring in diabetic patients, continuous tracking of inflammatory biomarkers for dry-eye disease, and around-the-clock intraocular pressure monitoring for improved glaucoma management.^33–36^

Despite these promising advances, significant challenges persist in achieving an optimal balance between biocompatibility, sensor performance, power efficiency, and user comfort. Minimizing optical background interference from tear-fluid constituents and maintaining long-term sensor stability during extended wear are critical obstacles for clinical translation. Addressing these hurdles requires innovations in materials science, surface engineering, and signal amplification methods, as well as the establishment of robust protocols for large-scale manufacturing and standardized clinical validation.

The journey toward integrating sensing elements into contact lenses began with the invention of the poly­(hydroxyethyl methacrylate) (pHEMA) hydrogel lens by Wichterle in 1970, transitioning the market from rigid poly­(methyl methacrylate) (PMMA) lenses to more comfortable and scalable soft hydrogels.^52^ Over subsequent decades, steady advancements in lens materials—particularly regarding biocompatibility and fabrication precision—laid the groundwork for embedding biosensors directly within contact lenses (Figure 1i). Around 2010, developments in microelectromechanical systems (MEMS), flexible electronics, and microfabrication enabled electrochemical sensing modules, typically relying on enzymes like glucose oxidase or lactate oxidase, to be embedded in lenses.^36,37,42,53^ Although enzyme-based systems demonstrated excellent analytical sensitivity, they faced inherent limitations, such as enzyme degradation, short operational lifetimes, and frequent recalibration requirements, alongside discomfort caused by integrated electronic components and power modules.^54,55^

**Figure 1:**
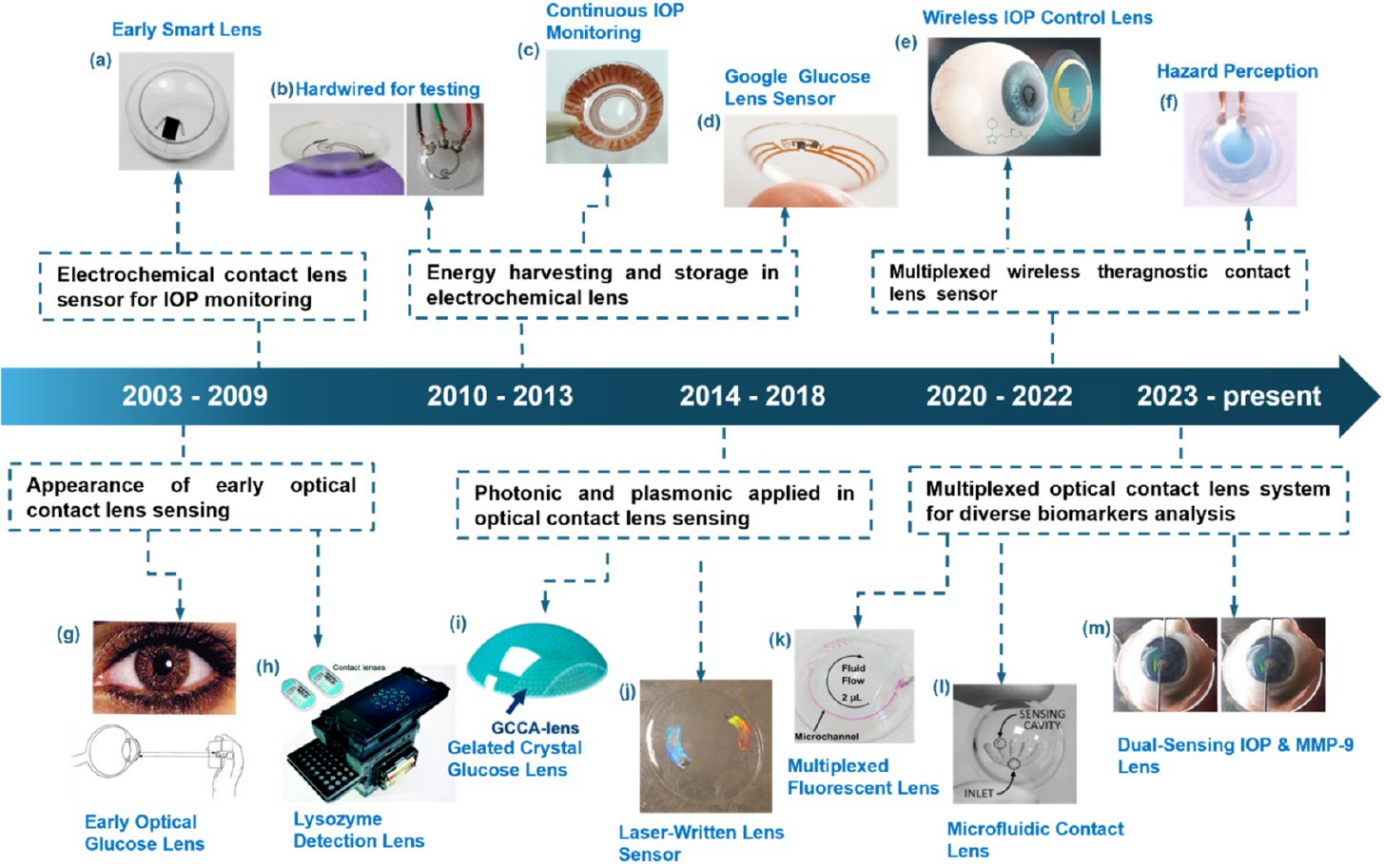
Timeline of contact lens sensor development. (a) Wireless contact lens sensor for intraocular pressure monitoring, Copyright 2008 Acta Ophthalmol.^38,39^ (b) Contact lens with embedded sensor for monitoring tear glucose level.^40^ Copyright 2011, with permission from Elsevier. (c) A capacitive contact lens sensor is used for continuous monitoring of intraocular pressure.^41^ Copyright 2013, with permission from Elsevier. (d) Google’s project for commercialized contact lenses for glucose sensing.^42^ Copyright 2017 American Chemical Society (e) Wireless theragnostic contact lens for monitoring and control of IOP.^43^ Copyright 2022 Springer Nature. (f) Contact lenses for hazard perception.^44^ Copyright 2020, with permission from Elsevier. (g) Early optical glucose-sensing contact lens.^45^ Copyright 2005, with permission from Elsevier. (h) Lysozyme detection in tears using a contact lens with a mobile sensor.^46^ Reproduced with permission from the Royal Society of Chemistry. (i) A gelated crystal-attached lens for continuous glucose sensing.^47^ Copyright 2017 MDPI. (j) Direct-laser writing of contact lens sensor.^48^ Copyright 2018 American Chemical Society. (k) Multiplexed fluorescent scleral lens sensor for tear ions measurement.^49^ Copyright 2019 A. K. Yetisen. Published by WILEY-VCH Verlag GmbH & Co., KGaA, Weinheim. (l) A paper-based microfluidic contact lens platform for tear pH, glucose, proteins, and nitrite ions sensing.^50^ Copyright 2020, with permission from Elsevier. (m) Contact lens dual-sensing platform for monitoring IOP and matrix metalloproteinases-9 (MMP-9).^51^ Copyright 2022. Advanced Science published by Wiley-VCH GmbH.

Optical sensing technologies provide real-time readout, high sensitivity (often nM to pM) and straightforward multiplexing, while avoiding the enzyme instability and hard-wired electronic interfaces typical of electrochemical designs (Figure 2). Techniques such as photonic crystals,^56,57^ holographic gratings,^58–60^ Förster resonance energy transfer (FRET)-based probes.^58,61^ and surface-enhanced Raman scattering (SERS) sensors enable label-free, real-time monitoring with exceptional sensitivity and multiplexing capabilities. By detecting subtle optical changes—shifts in diffraction wavelength, fluorescence intensity variations, or Raman scattering enhancement—these optical platforms can directly quantify low-abundance proteins, metabolites, electrolytes, and hormones within tear fluid, all without the instability issues inherent in enzyme-based methods or the complexity of wired electronic interfaces.^62,63^

**Figure 2:**
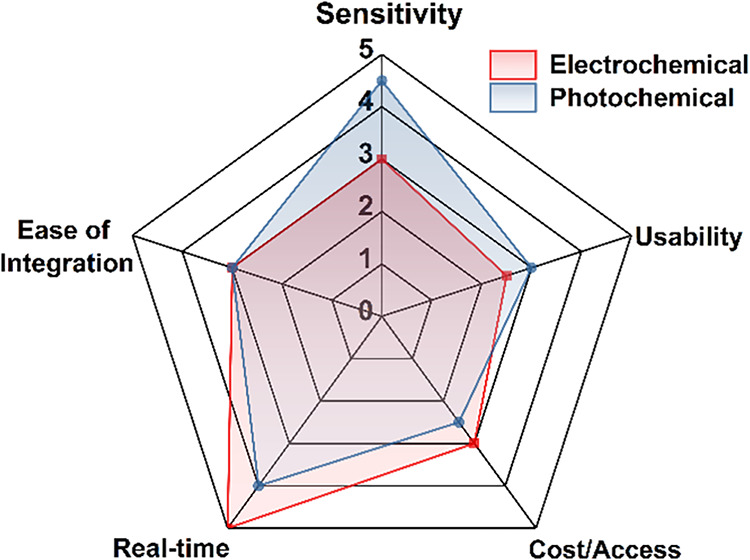
Electrochemical vs photochemical contact-lens sensing—trade-off summary. The radar plot shows normalized scores for sensitivity, usability, cost/accessibility, real-time feasibility, and ease of integration.^1,2^

Although substantial progress has been achieved, major engineering challenges remain in seamlessly integrating these advanced optical sensors into contact lenses. Achieving reproducibility and durability without compromising comfort, vision clarity is crucial for user acceptance. Rigorous clinical testing is also necessary to ensure sensor accuracy, robustness against fluctuations in tear film, and reliability in diverse environmental conditions. Furthermore, a comprehensive review highlighting recent advances in contact lens optical sensing materials, fabrication approaches, and integration methods remains notably absent from the current literature. This review, therefore, systematically examines optical biosensor integration from substrate selection—covering rigid, soft hydrogel, and hybrid lens materials—to detailed sensor embedding strategies within lens surfaces, intermediate layers, and internal microstructures. Lastly, we outline the path toward clinical translation, clearly delineating current technological gaps and future directions to realize contact lens-based diagnostics for real-time, point-of-care monitoring of ocular and systemic diseases.

## Tear Fluid-Based Biosensing for POC Diagnostics

Alternative sample types that can replace blood in noninvasive diagnostics have been the focus of intense investigation for over a decade.^64,65^ And can potentially be used for disease screening.^66^ In general, the tear film consists of an ultrathin lipid layer, a bulk aqueous layer, and an innermost mucin layer (Figure 3). The formed liquid barrier between the air and the proximal ocular tissue keeps the cornea moist and maintains the ocular antibacterial system.^67^

**Figure 3:**
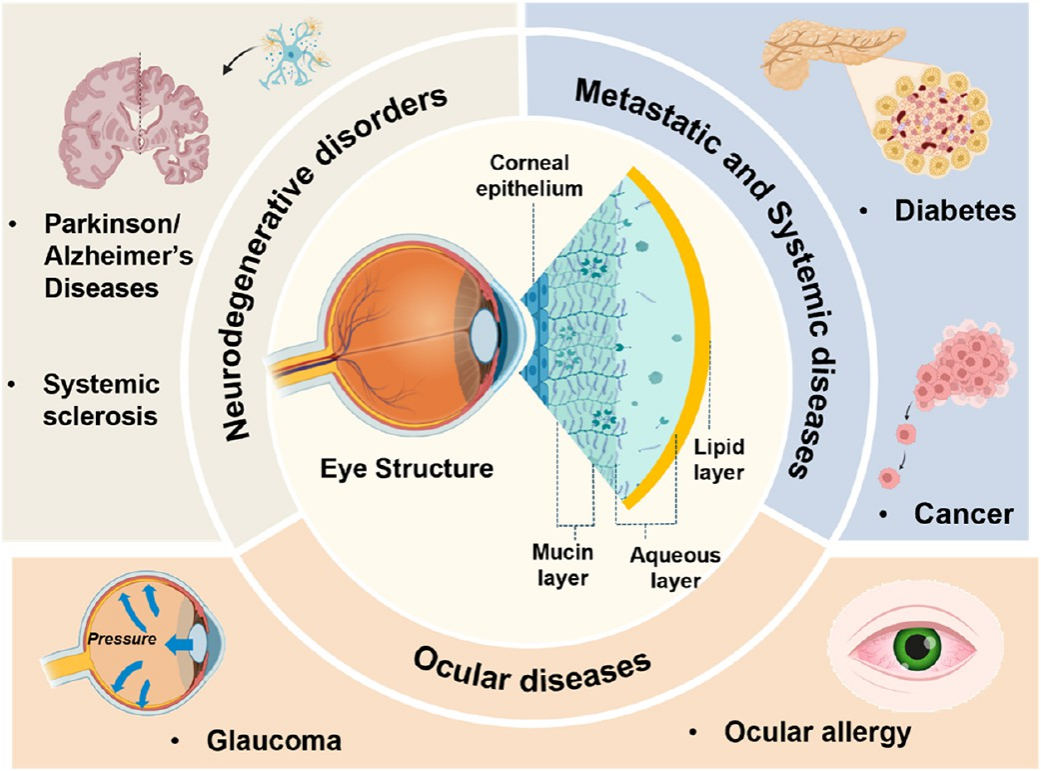
Tear fluid based biosensing for POC diagnostics. Schematic representation of the eye, tear layers, and four diagnostic categories (Neurological, Metastatic, Ocular, Systemic).

Tear fluid has demonstrated significant value in disease diagnosis and monitoring, extending beyond ocular pathologies. In healthy eyes, the blood-tear barrier restricts the movement of compounds, such as albumin and xenobiotics, between the tear film and blood. In diseased eyes, this barrier may be compromised, leading to increased permeability, greater systemic absorption, and reduced ocular drug bioavailability due to interactions with tear proteins. Research indicates that tear fluid closely resembles the ultrafiltrate of blood plasma. Mitochondrial energy metabolism and specific metabolic processes during plasma leakage facilitate the transfer of components from the blood to tears.^68,69^

Tear fluid’s relatively simple matrix compared to serum and plasma, together with its rich reservoir of biochemical and biophysical markers (Table 1), makes it an ideal medium for point-of-care diagnostics. Ocular conditions, including keratitis and allergic conjunctivitis, are readily identified through changes in tear analytes.^70,71^ While glaucoma monitoring has leveraged matrix metalloproteinases (MMPs) and their tissue inhibitors (TIMPs).^70^ Electrolyte profiles (K^+^, Na^+^, Ca^2+^) distinguish dry-eye subtypes such as lacrimal gland dysfunction (LGD) and meibomian gland dysfunction (MGD).^49^ Beyond ocular pathologies, systemic disorders are reflected in tear fluid: elevated glucose signals diabetes,^72^ increased lacryglobin correlates with cancer metastasis,^72,31^ and detectable TNF-α marks Parkinson’s disease.^73,74^ Complementary metrics—tear volume, moisture content, and intraocular pressure (IOP)—provide additional insight into ocular health.^75,76^

**Table 1:** Summary Table of Representative Tear Biomarkers, Associated Conditions, and Typical Concentration Ranges

category	biomarker	disease/condition	typical tear concentration	ref
neurological	α-antichymotrypsin	multiple sclerosis	∼1.2–2.3 μg/mL	^3^
	TNF-α	diabetic retinopathy (NPDR → PDR)	1.2–5.5 pg/mL (NPDR); up to 21.7 pg/mL (PDR)	^5^
metastatic	lacryglobin	breast and other cancers	detected in 60–100% patients	^31^
	sulf-1; AMPKγ3	cancer metastasis (proteomic study)	qualitatively detected (n.d.)	^32^
ocular	lysozyme C	general ocular surface health	∼1.4 mg/mL	^32^
	lactotransferrin	dry eye syndrome	∼1.0 mg/mL	^32^

Over the past decade, optical contact-lens biosensors have harnessed these tear biomarkers using fluorescence,^7,55,67,68^ colorimetric,^77–80^ and photonic-crystal^47,48^ platforms. They were fabricated focused on both paper- and lens-based substrates. These innovations have expanded the detectable panel and pushed detection limits into the low micromolar and nanomolar ranges, yet achieving high selectivity for low-abundance analytes while minimizing background interference remains a critical challenge.^81^ Future work must refine sensor chemistries and integration strategies to fully realize the promise of tear-based POC diagnostics.

## Contact Lens Materials

Contact lens sensors represent a significant advancement in wearable technology, merging the fields of optics and material science to enhance vision and health monitoring. These sensors are embedded into contact lenses using advanced materials tailored for specific properties. Typically, they employ flexible, ultrathin substrate materials that conform to ocular curvature, ensuring comfort and functionality. Additionally, the water content of the materials significantly impacts comfort, while mechanical properties influence durability and resistance to deformation during handling. Optical properties, including transparency and refractive index, are vital for effective vision correction. For medical applications, these lenses must also meet stringent regulatory standards while addressing the end wearer’s priorities for comfort, durability, and ease of use.^52,82^ The integration of biosensing capabilities further exemplifies how materials science should advance the functionality and versatility of contact lens sensors for noninvasive health monitoring and personalized medical care.^83^

Polymeric materials are widely used in the development of contact lens sensors, primarily due to their capacity for postfunctionalization that enhances sensing performance. These materials are crucial for integrating sensing technologies, enabling real-time monitoring of ocular conditions and the detection of tear biomarkers. Commonly used polymers in such applications include hydrogels and specialized polymers, chosen for their good biocompatibility and mechanical stability.^84–87^ In addition, these polymeric materials provide a structural matrix for drug delivery. This dual functionality supports not only biosensing but also the controlled release of drugs directly to the ocular surfaces, thereby maximizing therapeutic efficacy. These materials can also act as a protective barrier to shield the ocular surface from harmful substances while maintaining specific properties, such as oxygen and liquid permeability.

Proper lens curvature and thickness are essential to minimize irritating tear production and avoid localized inflammation.^88^ The microfabrication of sensing material always results in a shift of structural strength. A tensile strength of 0.3–1.5 MPa ensures contact lenses withstand mechanical stresses from insertion, removal, and blinking without permanent deformation. It is also essential to maintain a comfortable, nonirritating interface for adequate and consistent optical performance throughout daily wear.^89^ Current commercialized contact lenses can be divided into three main categories by softness: hard lenses, hybrid contact lenses, and soft contact lenses (Table 2). Although rigid contact lenses are less comfortable, their specific functions in correcting certain corneal irregularities, such as irregular corneal astigmatism and its associated diseases, cannot be ignored. Despite this, soft contact lenses are expected to dominate the commercial market due to their superior comfort and convenience for daily use.

**Table 2:** Comparison between Different Categories of Contact Lens Materials

	material	comfort	DK value (Barr)	EWC (%)	lens thickness	durability
hard lenses	PMMA	initially less comfortable, longer adaptation period	8–10	N/A	0.12–0.20 mm	2–3 years
	RGP		>30	N/A		
soft silicon lenses	SIMA	relatively comfortable, high oxygen permeability; low tolerance	10–30	38–70	0.07–0.1 mm	one day to half a year
	SIA					
	TRIS					
soft hydrogel lenses	HEMA	require short period for adaptation; short wearing period	60–150	30–60		
	PVA					
	MAA					
hybrid lenses	rigid center (RGP) with soft outer skirt (SiHG)	combines clear vision with comfort but costly	central: 10–60	50 for SiHG part	∼0.2 mm	depends on the hybrid materials
			peripheral: 60–160			

### Hard Lenses

The most widely used rigid contact lens material, as a substitute for traditional glass material, is PMMA, which was selected for its advantages of lightweight and durability.^90–92^ The intermolecular forces, including dipole–dipole interactions and mechanical interlocking inside the polymer structure, also led to superior rigidity. In addition, the weak mobility of the chains restricts the diffusion of water and oxygen, and prolonged wear may trigger corneal hypoxia.^93^ A market share of less than 1% proves the inappropriateness of lens materials.^94^ As an improvement, silicone acrylates were introduced in PMMA as rigid gas permeable (RGP) material to increase wearing comfort while retaining the inherent advantages of PMMA.

### Soft Lenses: Pros and Cons of Hydrogel vs Silicone Hydrogel

Soft contact lenses are made of hydrogels with high water content, which gives them exceptional flexibility. Cross-linking agents, such as ethylene glycol dimethacrylate (EGDMA), can enhance its mechanical properties and gelling, thereby maintaining a balance between structural integrity and oxygen permeability. As a result, they dominate the commercial contact lenses market due to their superior comfort and convenience in daily use. The two main soft lens materials include conventional hydrogels and soft silicone hydrogels.

#### Conventional Hydrogels

Hydrogel, including poly­(vinyl alcohol) (PVA) and poly­(2-hydroxyethyl methacrylate) (pHEMA), has a broad market application prospect. PVA is a relatively new synthetic polymer that offers the advantages of high biocompatibility and hydrophilicity due to the presence of a hydroxy group in its monomer, resulting in high tensile strength and low protein absorption for contact lenses. However, its low gas permeability, similar to that of PMMA, requires improvements for long-term comfort and eye health. Hydroxyethyl methacrylate (HEMA) is another popular material for commercial application; it is commonly copolymerized with monomers (e.g., Methacrylic Acid or *N*-Vinyl-2-pyrrolidone) to create pHEMA hydrogels, improving wettability and structural stability.^95,96^ Despite some incompatibilities with microfabrication technologies, pHEMA’s excellent transparency and mechanical properties make it ideal for advanced diagnostic and therapeutic contact lens applications.

#### Silicone Hydrogels

Contact lenses made of silicone hydrogel have already occupied the most significant contact lens market share due to their durability for long-term use, coming from the intrinsic and robust silicon–oxygen bonding.^97,98^ However, the lenses’ inherent hydrophobicity reduces their biocompatibility. For biosensing applications, Badugu et al. discovered that silicone hydrogel lenses have regions that interact with water-soluble molecules and hydrophobic zones that bind nonpolar molecules. These properties have been utilized in designing biosensors to detect various elements.^99–101^

### Hybrid Lenses

The hybrid lenses are primarily designed to have a central zone made of RGP materials with high optical properties and a peripheral zone made of silicone hydrogel, which is more comfortable to wear. The overall larger diameter of the hybrid lenses also brings the tendency toward the center. Moreover, problems exist, such as a tight fit in the peripheral segment and deposit formation.^9,10^ Breakage at the RGP/hydrogel junction was also reported to be as high as 48.5% of cases fitted with SoftPerm lenses.^6^ The optimized lens materials could not only deliver both comfort and clear vision but also define discrete regions for biosensor integration.

## Principles of Optical Sensing in Contact Lens

One of the key advantages of optical contact lens sensors is their ability to incorporate a range of sensing techniques enabled by a range of fabrication methods (Figure 4). Fabrication technologies, such as laser ablation, micromolding, microimprinting, and photolithography, allow for the precise integration of sensors within the lens architecture during contact lens production. By creating microfluidic channels inside the lens via micro molding or a laser ablation, changes in signals within specific areas can be more precisely localized and captured by portable readout devices.^50,102,103^ Additionally, microimprinting or direct modifications integrate fluorescence, colorimetric probes, or crystalline colloidal arrays (CCA) inside the substrate materials, providing overall signal variations and balance signal detection capabilities with minimal alterations to the material properties of the lenses.^76,104^

**Figure 4:**
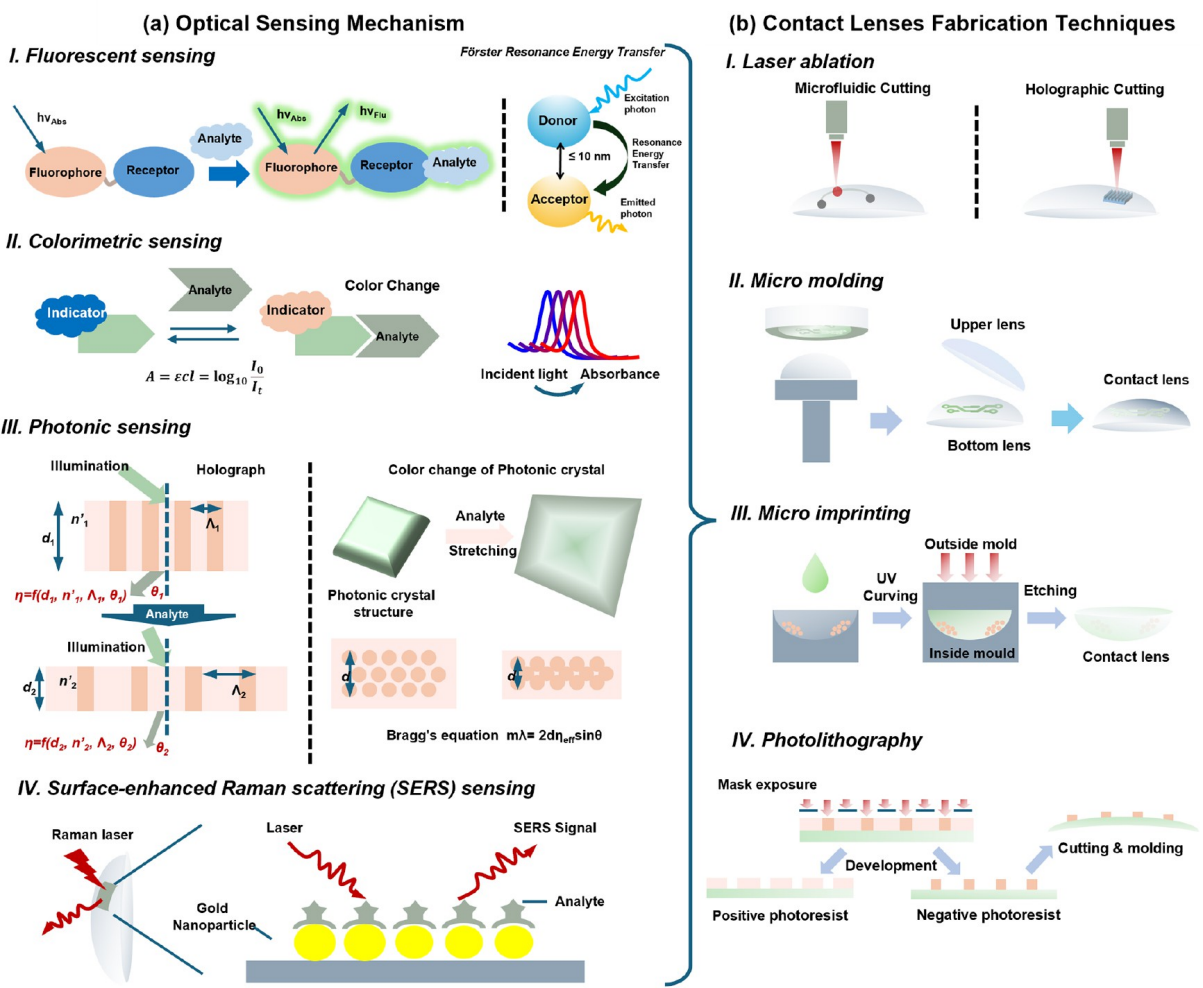
Assembly principle of a contact lens sensor. (a) Schematic of main optical sensing mechanisms: *I. Fluorescence sensing, II*. *Colorimetric sensing, III. Photonic sensing and IV. SERS sensing* in optical contact lens sensors. (b) Fabrication techniques to integrate the sensor in contact lens substrate materials including *I. Laser ablation, II. Micro molding, III. Micro Imprinting and IV. Photolithographic*.

As summarized in Table 3, each optical sensing modality offers distinct advantages and disadvantages specifically relevant to contact lens integration. Fluorescence-based lenses have demonstrated successful real-time glucose monitoring in diabetic patients, combining high sensitivity and rapid response, although the embedded optics can present integration challenges and potential discomfort.^105^ In contrast, colorimetric contact lens sensors provide simplicity and optics-free operation, making them user-friendly, though they sacrifice sensitivity and real-time capabilities.^76,106^ Photonic sensors enable multiplexed, label-free detection with high specificity but require complex microfabrication, increasing manufacturing difficulty and cost.^60,107^ Finally, SERS-based lenses have achieved ultrasensitive, low-abundance biomarker detection (down to picomolar levels), offering immense diagnostic potential, yet their implementation is hindered by complex nanostructured substrates and higher production costs.^108^ These examples collectively confirm the feasibility and versatility of integrating optical sensing modalities into contact lens platforms, each with trade-offs in sensitivity, complexity, cost, and real-world applicability. Consequently, essential health data is provided seamlessly without disrupting daily activities. Each optical modality has been validated beyond the bench, from diabetic-volunteer pilots and approved diagnostic assays to human wearables and clinical specimen tests. These confirm both their readiness for in vivo use and the feasibility of integrating them into contact-lens sensor platforms.

**Table 3:** Comparative Performance of Optical Sensing Modalities for Tear-Fluid Biosensing

modality	LOD	specificity	ease of Integration	clinical/field validation	ref(s)
fluorescence	glucose: 0.01 mM; Na^+^: 120 mM	high	moderate (requires optics)	human diabetic trial: tear-glucose lens readout <1 min, *R* ^2^ = 0.92 vs blood glucose in 8 volunteers	^109^,^110^
colorimetric	lactate: 0.03 mM; pH: ±0.1 unit	moderate	easy (embedded reagent)	sweat-chloride patch for cystic fibrosis: ≥98% sensitivity, at 60 mM cutoff	^111^,^112^
photonic	sweat glucose: 0.12 mM	high	challenging (microfabrication)	wrist-watch SPR sensor, *R* ^2^ = 0.95 vs blood glucose in *n* = 10 human subjects	^113^,^114^
SERS	SARS-CoV-2 N-protein: 5.2 PFU/mL	very high	difficult (nanostructure)	SERS-LFIA assay: rapid COVID-19 antigen test vs RT-PCR (*n* = 60 samples)	^108^

### Fluorescence Sensing

Fluorescence biosensors have become increasingly important due to their high sensitivity, specificity, ease of use, and relatively low cost.^115–117^ It readily reach nM−μM LODs for glucose and ions, making them ideal for metabolic monitoring in tears,^35,49^ with performance dependent on factors such as sensor status, detection range, and response time. Key principles for strong fluorescence include absorption at wavelengths that prevent molecular dissociation and a faster rate of radiation than intramolecular energy transfer. Advanced technologies, such as FRET, enable multianalyte monitoring within a single detection system, improving diagnostic accuracy for ocular diseases and enhancing clinical studies. FRET occurs when an excited donor fluorophore transfers energy to a nearby acceptor, resulting in decreased donor fluorescence intensity and lifetime while increasing acceptor emission. Unlike direct fluorescence detection, FRET remains unaffected by fluorophore concentration fluctuations, reducing interference from photobleaching and diffusion. Its ability to minimize light scattering enhances the accuracy of in vivo sensing. Moreover, fluorescence is highly molecule-specific as it depends on the chemical composition of the target fluorophore. This specificity makes FRET particularly effective for detecting tear biomarkers associated with conditions such as dry eye disease and diabetic retinopathy.

1 $$\mathrm{excitation}:,S_{0}+h\nu_{\mathrm{ex}}\to S_{2}$$

2 $$\mathrm{fluorescence}:,S_{2}\to S_{0}+h\nu_{\mathrm{em}}$$

Fluorescence in contact lens sensors has raised attention to their specificity and versatility. For example, with the advantages of assessing the severity of dry eye disease and differentiating its subtypes, studies have been performed to develop fluorescent contact lens sensors to monitor physiological levels of pH, Na^+^, K^+^, Ca^2+^, Mg^2+^, and Zn^2+^ ions, providing quantitative data via smartphone readouts.^99,118,119^ The continuous evolution of fluorescence sensing technologies holds significant promise for real-time ocular disease diagnosis and understanding of ocular physiology.

### Colorimetric Sensing

Colorimetric sensing leverages enzymatic reactions to achieve μM–mM detection ranges with high specificity. It is simple, reagent-based format can be readily implemented on paper- or lens-embedded platforms.^120,121^ Most of colorimetric contact lens sensors functioned based on the Beer–Lambert law, $A=\varepsilon \mathrm{cl}=\log_{10}\frac{I_{0}}{I_{t}}$ . In which *A* is the absorbance, ε represents molar absorptivity or the extinction coefficient, *c* is the concentration in the tear liquids, and *l* represents the path length of the sample, while *I*_0_ and *I_t_* are the intensity of incident and transmitted light separately. It quantificationally helps for the derivation of the concentration change in solution by measuring the absorbance of the incident light, accompanied by visible color change, which could also be specified and calculated in an RGB graph.

Riaz et al. proposed a dynamic ocular pH biosensor utilizing anthocyanins, which change chemical structure and color at different pH levels.^78^ Extracted from *Brassica oleracea*, anthocyanins were used to functionalize commercial soft contact lenses through soaking and drop-casting processes, with an optimized soaking time of 24 h. The sensors exhibited negligible dye leakage over 18 h and demonstrated a correlation between pH and color (measured via RGB triplets), indicating potential for continuous POC applications.^78^

### Photonic Sensing

Photonic crystals (PCs) are composed of periodically ordered materials with varying refractive indexes based on the direction of periodicity and are classified into one- to three-dimensional (1–3D) structures. When illuminated by polychromatic light, these crystals diffract light according to Bragg’s law. Accordingly, the diffracted wavelength changes due to any modification in the periodic constant or the effective refractive index. Subnanometer changes in lattice spacing or refractive index yield a pronounced diffraction-peak shift. Alexeev et al. demonstrated a detection limit of ≈1 μM glucose in synthetic tear fluid, with diffraction peaks shifting by 10 nm over a 0.1–0.6 mM glucose range.^122^

Photonic sensors have been integrated into contact lenses as forms of holographic gratings or CCA.^47,122–127^ Generally, 1D photonic structures are fabricated utilizing deposition or laser ablation, while 2D and 3D structures are obtained by photolithography or microimprinting.^59,60,128,129^ The diffraction of light derived from structural change indicates minimal alteration on the PC plane, allowing for the quantification of tear analytes through color variations. Multiple studies have developed contact lens sensors with holographic gratings to test glucose in tear fluid at various physiological pH conditions and ionic strengths.^130,131^

3D photonic crystal array sensors are particularly notable, comprising nanosized particles immobilized within polymer matrices. These arrays are formed from crystalline nanospheres, such as polystyrene and silica, that self-assemble into ordered structures upon the evaporation of colloidal solutions. The photonic CCA-based sensors are embedded within a polymer matrix that diffracts light in the visible spectrum. Such advanced PCs have been widely investigated for tear-based diagnostics, offering precise and responsive sensing capabilities. The dye-free nature of photonic sensors avoids the use of dyes and keeps them away from photobleaching, resulting in a longer use time.

### Surface-Enhanced Raman Scattering (SERS) Sensing

Surface-enhanced Raman scattering (SERS) amplifies intrinsically weak Raman signals at plasmonic “hot spots” formed near nanostructured Au/Ag features, providing label-free molecular fingerprints with ultrahigh sensitivity (down to the pM regime) and rapid spectral readout.^132,133^ On contact lenses, SERS elements are typically introduced by surface transfer/stamping of prepatterned metallic films or by in situ immobilization of nanoparticles, nanoislands, or nanobowls within the hydrogel matrix. These strategies localize intense electromagnetic fields at the tear–lens interface, enabling detection of low-abundance biomarkers under physiologically relevant conditions. Using such architectures, proof-of-concept lenses have quantified glucose with high sensitivity, and related platforms have reported SERS readout of proteases/cytokines at clinically meaningful concentrations.^78,134–136^

Design considerations strongly influence performance and safety. Plasmonic resonance tuning (particle size/shape, intergap spacing, lattice periodicity) is matched to NIR excitation to reduce hydrogel autofluorescence and ocular absorption, while thin dielectric shells and PEG/zwitterionic coatings mitigate ion leaching, nonspecific adsorption, and biofouling in the protein- and salt-rich tear film.^137–139^ Stable anchoring chemistries, including thiol–Au, catechol–metal, UV-cross-linked interpenetrating networks, prevent nanoparticle migration during wear, and peripheral or annular SERS zones help preserve pupil optics and wearer comfort. From a manufacturing standpoint, SERS features must remain compatible with sterilization (UV/EtO/γ) and saline storage without loss of enhancement, while maintaining hydrogel transparency and modulus.^140–142^

## Optical Readout and Mitigating Interference of Contact Lens Sensor

Readout options for contact-lens sensors include fluorescence, colorimetric, photonic/holographic, and SERS, each with practical constraints: fluorescence and SERS are reliable with brief ex vivo interrogation; colorimetry supports low-cost and fast imaging; photonic/holographic diffraction remains alignment-sensitive.

### Smartphone Integration in Fluorescence Readout

Signals from fluorescence-based contact lens sensors are typically captured using either compact benchtop fluorimeters or smartphone cameras combined with a custom-designed, 3D-printed lens holder. In most current setups, lenses are briefly removed (<1 min) and placed within holder, where they are illuminated by a low-power LED with an appropriate excitation wavelength.^62,143^ An integrated optical filter positioned between the lens and detector isolates the fluorescence emission, reducing background interference and enhancing signal clarity. The emitted light is captured by the smartphone camera or a small photodiode detector, and the resulting signal is analyzed by dedicated software or smartphone apps to quantify biomarker concentration. However, this approach introduces additional complexity, increased power demands, and higher costs, requiring careful optimization to ensure practical usability and comfort.

### RGB Imaging for On-Lens Assays in Colorimetric Readout

Color changes in colorimetric contact lens sensors can be read by eye for semiquantitative use or precisely quantified using smartphone apps that analyze RGB values. Because no external optics are required, users can remove the lens for a 10 s photo, making this the cheapest and most accessible modality.^144,145^

### Holographic Readout: Alignment-Sensitive Readout

Reading out of photonic-based lenses currently relies on external spectrometers or smartphone attachments, as precise optical alignment and careful calibration remain challenging. While proof-of-concept goggles integrating on-eye readout optics have been demonstrated experimentally, practical limitations in alignment precision, complexity, and cost have largely confined photonic sensors to clinical or research environments rather than routine home use.^60,146,147^

### SERS Readout: Practical Constraints

Current implementations rely mainly on brief ex vivo interrogation: the lens is removed and placed in a holder on a benchtop Raman system to ensure stable excitation, precise alignment, and high detector sensitivity.^132–134^ Signal processing, including baseline correction and drift compensation, can further enhance specificity in the presence of eye motion and tear-film fluctuations.^148,149^ At present, SERS contact lenses are suited to high-sensitivity spot assays and clinical tear-specimen testing; continuous on-eye monitoring remains a future objective depending on stability and motion-robust quantitative spectroscopy.

### Practical Multiplexing and Interference Management

For multianalyte operation in tears, optical contact-lens sensors limit spectral crosstalk and matrix effects by selecting probe sets with well-separated excitation and emission bands. It use ratiometric or inner-reference calibration to suppress illumination and thickness drift, partitioning chemistries into discrete microdomains or microfluidic reservoirs to prevent cross-diffusion. Image-based chemometric analysis can be applied to unmix residual overlap. Selective receptor design further enhances specificity; tetrahedral boronated receptors for glucose minimize lactate interference and mitigate pH dependence across the tear range.^77,99,150,151^

In fluorescence sensing, dual-dye ratiometric schemes on silicone-hydrogel lenses have enabled concurrent readouts of pH, chloride, and sodium, while maintaining robustness to illumination fluctuations and thickness nonuniformity.^99,152^ In photonic and holographic designs, glucose-responsive systems using tetrahedral boronate receptors show attenuated lactate cross-reactivity and moderated pH response, and the use of a fixed reference band allows relative wavelength-shift calibration under tear-like conditions.^29,77,153^ In colorimetric microfluidics, segregated reaction reservoirs with smartphone-based RGB quantification physically isolate assays and limit diffusion crosstalk, enabling clean multiplexed measurements on-lens.^77^ In SERS platforms, the intrinsic narrowness of Raman bands provides naturally separable channels for multiple targets, though stable baselines, background suppression, and reliable spot alignment remain essential; most current demonstrations therefore perform a brief ex vivo readout after lens removal to ensure excitation stability and detector sensitivity.^51,154^

## Integration of Contact Lens Sensors

As an ideal platform for precise monitoring of biomarkers in tear fluid, contact lens sensors are required to ensure effective lens-ocular surface interaction and tear collection. The sensing techniques utilized, accompanied by the choice of substrate materials and constructions, have a significant effect on the sensors’ detection range and response time, with representative studies in recent years summarized in Table 4.

**Table 4:** Principal and Performances of Contact Lens Sensors

sensing mechanism	lens materials	analytes	bioactive molecule	detection range	limit of detection	response time	ref(s)
fluorescence	PVA	glucose	boronic acid containing fluorophores (BAFs)	50–500 μM	–	–	^105^
	pHEMA	Na^+^	diaza-15-crown-5 (DA15C5)	0–100 mM	15.6 mM	–	^118^
		K^+^	diaza-15-crown-5 (DA18C6)	0–50 mM	8.1 mM	–	
		Ca^2+^	1,2 bis(*o*-aminophenoxy) ethane-*N*,*N*,-*N*′,*N*′-tetraacetic acid (BAPTA)	0.5–1.25 mM	0.02–0.05 mM	–	
		Mg^2+^	5-oxazolecarboxylic acid (5OACA)	0.5–0.8 mM	0.10–0.44 mM	–	
		Zn^2+^	luorescent *N*-(2-methoxyphenyl) iminodiacetate (MPIDA)	10–20 μM	10–20 μM	–	
		H^+^	benzenedicarboxylic acid (BDCA)	pH 7–8	pH 0.12	–	
	SiHG	Na^+^	sodium green	–	0.3 mM	–	^119^
	SiHG	Cl^–^	SPQ-C18	–	10 mM	–	^99^
		H^+^	6HQ-C18	–	pH 6.5–7.0	–	
		polarity	NBD-C18	–	–	–	
	Nelfilcon A	glucose	tetradamine isothiocyanate–concanavalin A (Tritc_Con A)	–	–	–	^155^,^156^
	SiHG	glucose	Quin-C18	–	<100 mM	–	^100^
	pHEMA	glucose	GS-NHS	0.1–1 mM	9.3 μM	3–5 s	^157^
	pHEMA	glucose	cerium oxide nanoparticles	0.1–0.6 mM	0.1 mM	–	^158^
	SiHG	ascorbic acid	bovine serum albumin (BSA-AuNCs)	0.23–0.8 mM	0.23 mM	10–13 s	^63^
	SiHG	lactoferrin	trivalent terbium (TbCl^3^)	0.44–5 mg mL^–1^	0.44 mg mL^–1^	–	^62^
	SiHG	lysozyme	*Micrococcus lysodeikticus*	–	1.99 μg mL^–1^	10 min	^46^
colorimetric	pHEMA	glucose	3,3′,5,5′-tetramethylbenzidine (TMB)	1.1–10.0 mM	1.1 mM	15 s	^106^
		protein	3′,3″,5′,5″-tetrachlorphenol-3,4,5,6-tetrabromsulfophthalein	1.1–8.0 mg mL^–1^	1.1 mg mL^–1^	15 s	
		l-ascorbic acid	phosphomolybdic acid	0.059–1.0 g L^–1^	0.059 g L^–1^	25 s	
		nitrite	sulfanilamide	19.2–160 μM	19.2 μM	20 s	
	pHEMA	glucose	cerium oxide nanoparticle-poly(ethylene glycol)-glucose oxidase	–	<0.11 mM	10 min	^104^
	RGP	corneal temperature	cholesteryl oleyl carbonate (COC), cholesteryl nonanoate (CN), and cholesteryl benzoate (CB)	29–40 °C	–	490 ms	^159^
	pHEMA	exosome	CD81 antibodies	–	2.14 μg mL^–1^	–	^34^
	pHEMA	corneal temperature	chromogenic material	1–4 kPa	0.18 kPa	–	^160^
	pHEMA	IOP	–	33–38 °C	–	–	^76^
	pHEMA	timolol	–	–	–	12 h	^161^
photonic crystalline	PCCA	glucose	4-acetamido-3-fluorophenylboronic acid	–	10 μM	–	^122^
	GCCA	glucose	diols and borate ions	–	0.05 mM	–	^47^
	NIR-PCCA	glucose	phenylboronic acid	–	0.006 mM	–	^123^
	PCCA	DSP	phenylboronic acid	–	–	–	^60^
	PVA	glucose	phenylboronic acid	–	–	–	^128^
	PDMS	glucose	phenylboronic acid	–	0–50 mM	<30 min	^107^
SERS	SERS-LM	glucose	4-mercaptophenyl boronic acid (MPBA)	500 nM–1 mM	211 nM	–	^162^
	scleral lens	protein	parylene-C	–	–	–	^163^
	pHEMA	metabolic	SERS	–	–	–	^136^

### Surface-Mounted Optical Sensors on Contact Lens

Surface mounting uses coating or ablation to apply sensors directly, allowing postproduction modifications. The characteristic of direct contact with tear fluid also improves rapid analyte detection. Despite these benefits, sensors fabricated on the lens surface may be more vulnerable to mechanical abrasion or detachment during wear. The challenge lies in ensuring that the surface-adhered sensors remain stable and functional throughout the lens’ lifespan while maintaining the necessary biocompatibility and comfort for the wearer.

#### Dip-Coat or Spray-Coat Plasmonic/Holographic Nanofilms

Surface mounting attaches sensor films or patterns directly onto the lens exterior, offering straightforward fabrication and immediate tear contact. A contact lens sensor was developed by the fabrication of a plasmonic material prepared to transform glucose monitoring.^162^ This innovation utilized a layer-by-layer structure, beginning with a biocompatible silk fibroin (SF) layer that doubles as a molecular filter for the screening of the protein-rich milieu of human tears (Figure 5a,b). FE-SEM and FIB cross sections confirm AgNWs on the SF layer and the layered SERS-LM architecture. Atop this selective barrier lies a layer of silver nanowires (AgNWs), which was functionalized with 4-mercaptophenylboronic acid (MPBA) to form a dense matrix of hotspots for signal amplification. These hotspots, essential for enhancing Raman signals, are meticulously arranged, as revealed by FE-SEM images, thus providing a real-time window into the wearer’s glycemic state. The device’s precision stems from the cis-diol complexation between MPBA and glucose, where a protective film further protects the reaction from environmental interference.^162^ With a detection limit of 211 nM, this multilayer SERS-LM exhibits high sensitivity; the lens-mounted prototype (Figure 5c) and concentration-dependent Raman responses consistent with 4-MPBA glucose selectivity (Figure 5d) support its potential for tear-glucose monitoring and integration into wearable biofluid-sensing systems. Another smart contact lens tailored for the continuous monitoring of glucose in real-time physiological settings was proposed by Elsherif et al. (Figure 5e–g). This innovative glucose sensor integrates 3-(acrylamide) phenyl boronic acid (3-APB) within a polyacrylamide hydrogel matrix to form a highly responsive hydrogel.^60^ By employing replica molding, a 1D grating, acting as a diffraction element, was precisely engineered onto the hydrogel, which is ultimately attached to the surface of the commercial contact lens. Fabrication uses a polystyrene (PS) master as a stamp; monomer is drop-cast and UV-polymerized on the lens to form the imprinted photonic structure. This 1D grating acts as the optical transducer, converting hydrogel volumetric changes into discernible diffraction signals. Incorporation of the grating increases the sensor’s surface-to-volume ratio and hydrophobicity, enabling rapid response (∼3 s) and saturation (∼4 min) under continuous monitoring. The device was seamlessly integrated onto a commercial contact lens for tear-glucose detection, with a smartphone’s ambient light sensor used for readout. Consistent with this design, Figure 5f shows transmitted diffraction patterns at low glucose, cross-sectional changes versus glucose, and the projection/measurement setup, while Figure 5g illustrates glucose–phenylboronic-acid complexation in the 1D sensor.

**Figure 5:**
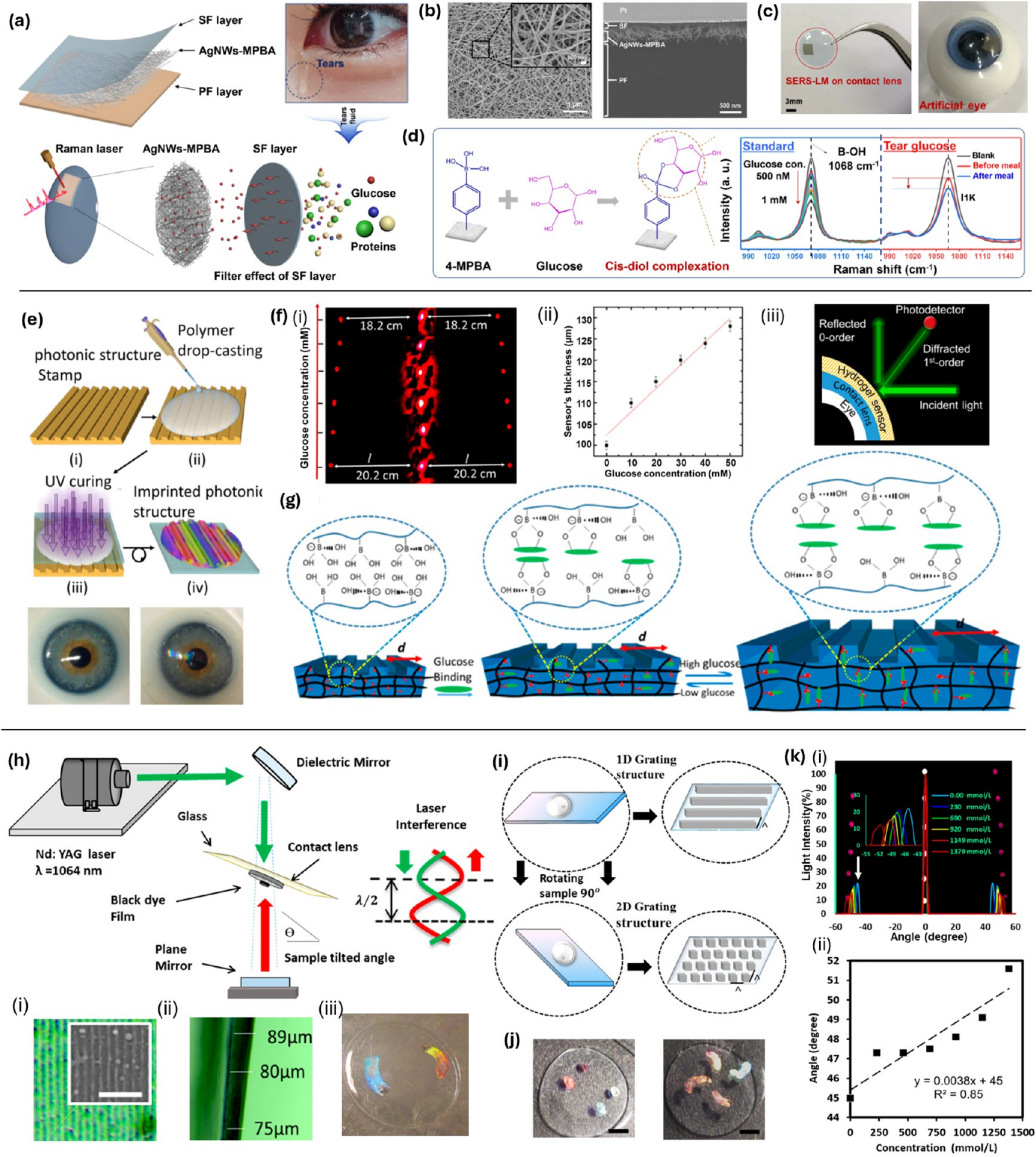
Surface-mounted optical sensors integrated onto the surface of contact lenses. (a) A combined SERS-LM structure integrated with a contact lens for selective glucose detection. (b) FE-SEM images show AgNWs on the SF layer, inset high-resolution nanowires, and SERS-LM cross-section after FIB cutting. (c) A prototype of the SERS-LM integrated into a contact lens. (d) The chemical selectivity of 4-MPBA for glucose with Raman spectra changes of the SERS-LM after reacting with varying glucose concentrations.^162^ Copyright 2020, with permission from Elsevier. (e) The fabrication of a hydrogel glucose sensor: I. a PS master is used as a stamp. II. PS is coated with monomer solution via drop-casting. III. UV polymerized monomer on contact lens. (f) i. Transmitted diffraction patterns of the PS sensor at low glucose concentrations are depicted. ii. The sensor’s cross-section changes versus glucose concentration. iii. The setup for projecting diffraction patterns and measurement. (g) Glucose–phenylboronic acid complexation in the 1D PS sensor.^60^ Copyright 2018 American Chemical Society. (h) One-dimensional nanopatterns on contact lenses are fabricated via DLIP with Nd laser (1064 nm, 3.5 ns). i. Optical microscopy of the 1D nanostructure with SEM (scale bar = 5 μm). ii. Lens cross-section in ambient humidity (scale bar = 100 μm). iii. Ink-based holographic nanostructures on lenses (scale bar = 5 mm). (i) 1D and 2D nanostructures are presented. (j) Holographic nanostructure designs (rings/patches) on contact lenses. (k) i. Diffraction measurements on nanopatterned lenses at different Na^+^ concentrations. ii. Diffraction angle variations corresponding to Na^+^ concentration changes.^48^ Copyright 2018 American Chemical Society.

#### Laser Direct-Write of Holographic Gratings

Another innovation in surface integration was achieved by directly writing nanophotonic structures onto contact lenses using laser technology. They deliver dye-free, real-time color shifts but require precision alignment and robust adhesion to the curved lens surface. In this study, 1D and 2D nanostructures were inscribed onto hydrogel contact lenses by employing a precisely controlled neodymium-doped yttrium aluminum garnet (Nd: YAG) laser beam to etch detailed grating structures into the lens material (Figure 5h–k).^48^ One-dimensional nanopatterns were fabricated by DLIP with an Nd laser (1064 nm, 3.5 ns), with optical microscopy/SEM of the nanostructure and a lens cross-section in ambient humidity. The precision of these nanostructures was measured using SEM images. The adaptability of this method is further highlighted through the creation of diverse holographic designs, such as rings and patches, which adapt dynamically to changes in Na^+^ ion concentrations, as evidenced by their diffraction patterns. Advancing this technology, the study introduces SERS functionality to the lens surface, which is particularly notable for its glucose detection capabilities. Postnanofabrication hydrophobicity changes indicate effects on surface properties relevant to comfort and wearability.

Despite these advancements, the sensitivity of smart contact lens sensors remains insufficient for accurately detecting glucose levels within the physiological range of tear fluid. Another challenge is the nonselective binding of boronic acid derivatives to other carbohydrates and hydroxyl acids, such as lactate, which can exhibit similar concentrations to glucose, leading to potential measurement inaccuracies. To mitigate this issue, the mole fraction of 3-APB was optimized to approximately 20 mol %, significantly enhancing the sensor’s selectivity for glucose and improving measurement reliability.^164^

### Physically Embedded Optical Sensors in Contact Lens

The embedding of optical sensors within contact lenses represents a classical approach in the field of ocular sensors, primarily employing fluorescence and colorimetric sensing mechanisms. In these cases, physical cavities or channels are created within the lens to accommodate various sensor elements. This fabrication method provides a protective environment for the sensors, potentially extending their operational lifespan and shielding them from direct exposure to the external environment. Embedded optical sensors facilitate multianalyte detection by integrating various sensing elements within a single lens. However, this may increase lens thickness or alter permeability, potentially affecting wearer

comfort. Balancing sensor integration with minimal lens modification remains a critical challenge in optimizing performance and user experience.

#### Laser-Ablated/Micro-Molded Microfluidic Channels

Microfluidic grooves carved by laser or micromold can house paper-based colorimetric strips or liquid reagents. This approach shields sensitive elements yet increases lens thickness and may reduce oxygen permeability.

An initial demonstration combined fluorescence sensing with microfluidics: bovine serum albumin–gold nanoclusters embedded in a PVA–citric acid film were cast onto a laser-ablated contact-lens substrate to monitor tear ascorbic acid (AA), a marker of ocular inflammation, in real time (Figure 6a–b).^63^ AA restores fluorescence quenched by KMnO_4_, yielding a linear response from 0 to 1.2 mM (LOD 0.178 mM), while laser-ablated microchannels direct tear flow across the sensing film. A custom 3D-printed smartphone cradle and companion app capture and quantify emission at room temperature (Figure 6c), and the sensor maintains stable performance over 20 h of use and 10 days of storage. A simple readout box fixes the region of interest (ROI) and labels each pad by analyte to standardize on-eye imaging (Figure 6j).

**Figure 6:**
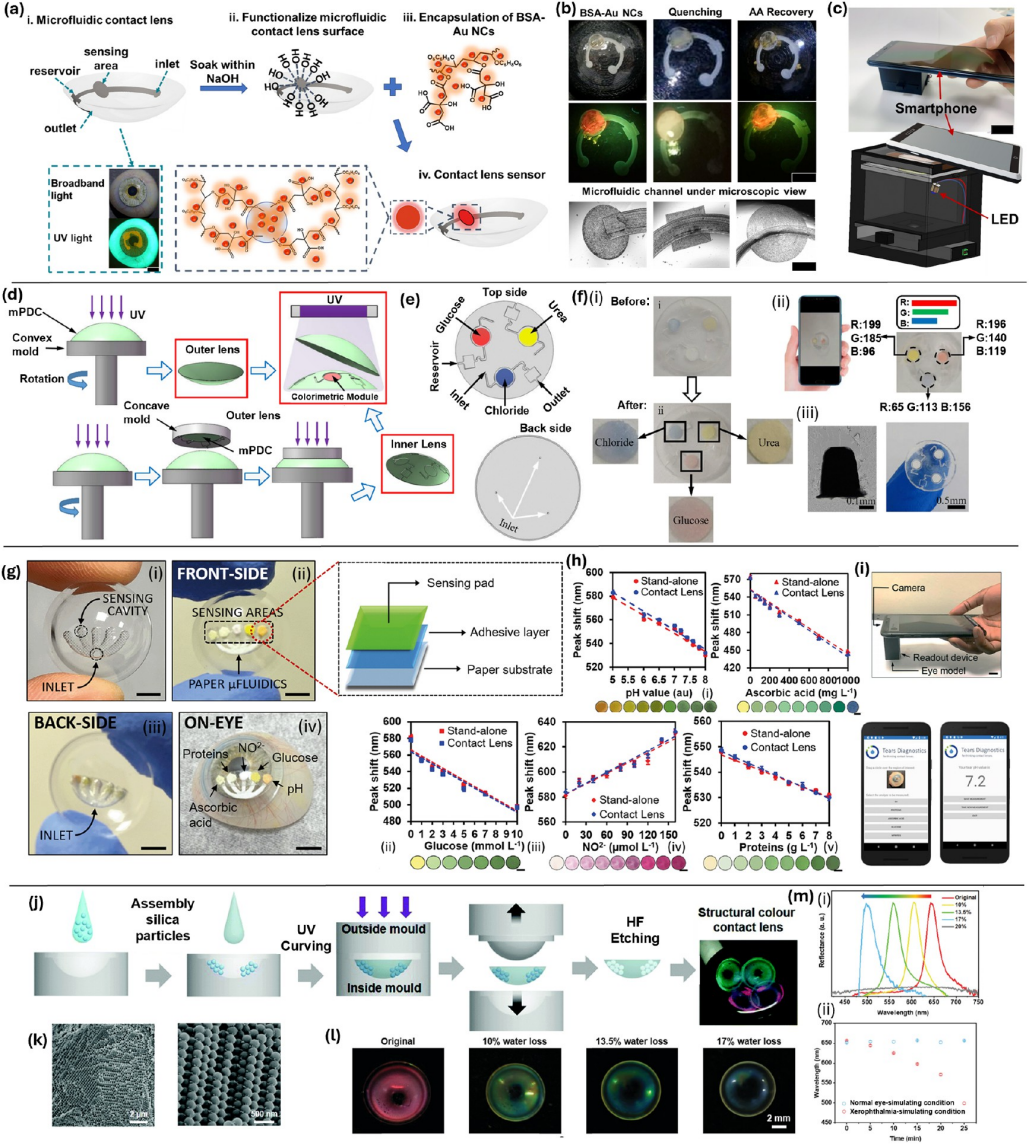
Fluorescent and colorimetric sensors physically embedded in contact lenses. (a) Implementation of BSA-Au NCs on microfluidic contact lenses: i. Laser-ablated microfluidic contact lens and vision site under broadband and UV light; ii. Functionalize contact lenses in 1 mol L^–1^ NaOH; iii. Encapsulate BSA-Au NCs with 15 wt % PVA and 1.5 wt % CA on the sensing region; iv Contact lens sensor for AA detection (scale bar: 2 mm). (b) Encapsulated microfluidic contact lenses and microscopic images of channels. (c) Design and photographs of the readout box for AA sensor.^63^ Copyright 2024 Published by Elsevier B.V. (d) Fabrication process of microfluidic contact lenses. (e) Top and back sides of the inner lens. (f) i. Color change before and after tear detection; optical photos and RGB levels for glucose, chloride, and urea concentrations; ii. Procedure for harvesting tears and color analysis of digital images; iii. SEM images of the microchannel cross-section and photo of a microfluidic contact lens.^103^ Copyright 2020, Springer Science Business Media (g) Device fabrication of microfluidic contact lens sensor: i. Laser-inscribed microfluidic; ii. Embedding paper microfluidic chip; iii. Backside view; iv. Contact lens on an artificial eye model (scale bars: 1.5 cm). (h) Reflection peak shift between stand-alone and contact lens-embedded sensors for i. pH sensor (range 5.0 to 8.0); ii. Ascorbic acid sensor (0 to 1.0 g L^–1^); iii. Glucose sensor (0 to 10.0 mmol L^–1^); iv. Nitrite sensor (0 to 160.0 μmol L^–1^); (v) Protein sensor (0 to 8.0 mg mL^–1^) (scale bar of insets: 1.5 mm). (i) Readout box for region of interest (ROI) and corresponding analyte.^50^ Copyright 2020, with permission from Elsevier. (j) Structurally colored contact lens sensors fabrication using a colloidal crystal template of monodispersed silica particles. (k) SEM images of the sensor with embedded colloidal crystal templates. (l) The structurally colored lens sensor and its color change with different water loss percentages. (m) i. Reflectance spectra of the red lens sensor with varying water loss percentages. ii. Plot of wavelength changes of reflectance peaks over time under different conditions.^76^ Reproduced with permission from the Royal Society of Chemistry.

Building on this microfluidic platform, a flexible, micromolded contact lens routes tears via capillary-driven channels from a peripheral inlet into central reservoirs preloaded with reagents for glucose, chloride, and urea (Figure 6d,e).^103^ Fabricated by micro-PCR molding to define precise curvature and channel depth, the lens ensures uniform flow. Colorimetric reactions then produce hue shifts proportional to analyte concentration, which are imaged by a smartphone (Figure 6f) and analyzed via RGB-to-concentration algorithms for multiplexed tear diagnostics.

More recently, CO_2_ laser ablation has been used to inscribe microchannels and biosensor-embedded microcavities directly into commercial contact lenses (Figure 6g–h). When tested with artificial tears, these devices exhibit rapid response times and high sensitivity across multiple analytes; data are processed through a smartphone-MATLAB interface (Figure 6i). Comparative measurements show consistent reflection-peak shifts for stand-alone versus lens-embedded formats across pH, ascorbic acid, glucose, nitrite, and protein.^50^ Together, these approaches illustrate a progression from fluorescence to colorimetric to laser-fabricated sensing architectures, each offering unique advantages for on-eye tear analysis in both clinical and point-of-care settings.

#### Replica-Molded Photonic Structures

Replica molding embeds 1D/2D photonic gratings directly into the hydrogel by pressing a nanostructured master (e.g., colloidal polystyrene or silica arrays) into the prepolymer solution, UV-curing, and demolding. Within the hydrogel contact lens domain, a pHEMA lens exhibiting colorimetric responses to intraocular pressure (IOP) changes represents a significant advance.^76^ The sensor is constructed by strategically assembling the hydrogel into a periodic structure using a dual-mold system followed by UV curing (Figure 6j). Unlike traditional pigmented sensors, this lens utilizes a 3D periodic structure that diffracts light based on its refractive index and the spacing of its lattice structure, causing visible color shifts correlated with IOP variations (Figure 6k).^76^ SEM confirms the embedded periodicity inherited from the colloidal-crystal template, and reflectance spectra track wavelength shifts with water-loss percentages and over time. When exposed to pressures ranging from 0 to 35 kPa, the lens exhibits a pronounced blue shift across the visible spectrum, demonstrating high sensitivity and precision in pressure detection. With a detection limit within the clinically relevant range, the sensor shows potential for monitoring intraocular pressure (IOP) in conditions such as glaucoma. Its capability to detect subtle pressure variations is supported by a strong linear correlation between wavelength shifts and pressure changes, attributed to alterations in the lattice spacing of its periodic structures (Figure 6l,m).^76^ This highlights its potential as a noninvasive diagnostic tool for ocular health monitoring.

### Microstructural Modification of Sensors inside Contact Lens

The chemical immobilization of optical sensors in contact lenses has ushered in a new wave of sensor development. This sophisticated approach involves incorporating fluorescent dyes or other sensing probes directly into the hydrogel matrix of the lens, such as pHEMA. Unlike other methods, this technique allows for a seamless and intimate interaction between the sensor and the analyte, resulting in heightened sensitivity and swift response. By manipulating the material at the molecular level, this method integrates the sensing function into the very structure of the contact lens without altering its inherent properties, such as comfort and transparency.

#### Physical Entrapment of Nanocluster Probes

Physical entrapment of nanoclusters leverages the hydrogel’s mesh to confine particle-based probes without chemical modification. Such molecular integration is particularly advantageous for continuous monitoring and has shown promising potential in glucose detection in tear fluid. A wearable glucose-sensitive fluorescent contact lens sensor represents a breakthrough in noninvasive glucose monitoring.^157^ By integrating a glucose-specific fluorescent probe alongside a reference dye for calibration within a hydrogel matrix, this sensor achieves highly sensitive glucose detection directly from the contact lens environment, with the detection ranging from 0.023 to 1.0 mmol L^–1^ through an evident color shift from pink to blue (Figure 7a–c). Further underscoring its clinical viability, in vivo experiments have confirmed the biocompatibility of the sensor with the rabbit model. The sensor’s detection threshold reaches 9.3 μmol L^–1^ when measured by a fluorescence spectrophotometer. This sensitivity not only enables continuous real-time monitoring of tear glucose but also distinguishes between nondiabetic and diabetic glucose levels.^157^ A smartphone application was finally designed to facilitate the transfer and interpretation of these glucose-induced variations (Figure 7d).

**Figure 7:**
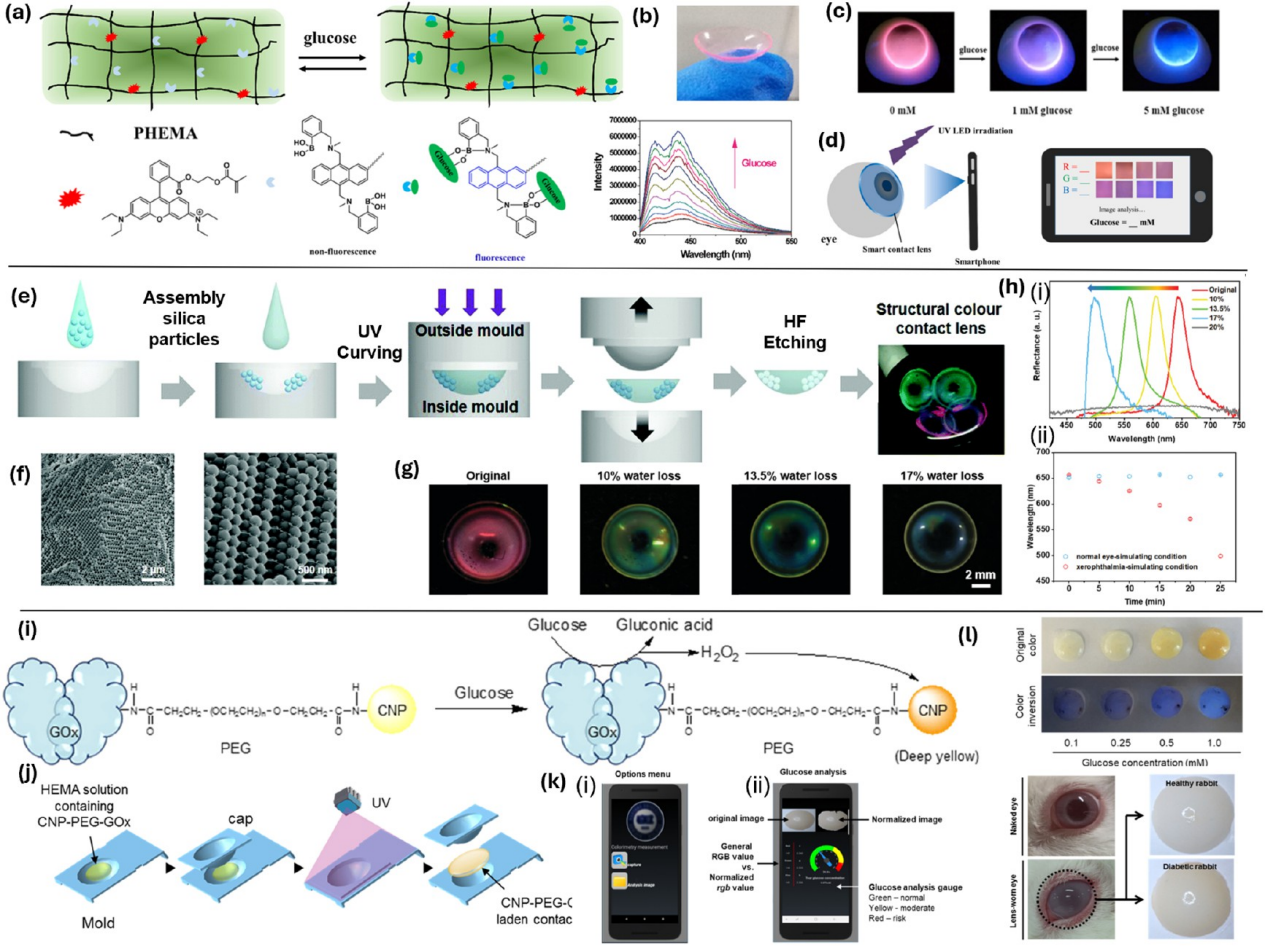
Chemical immobilization of optical sensors within the contact lens matrix. (a) The pHEMA hydrogel network contains a sensitive glucose probe and a calibration reference; adding glucose increases blue light emission, changing the lens fluorescence from red to blue. (b) Photograph of the soft, transparent smart contact lenses. (c) Fluorescent images of contact lenses with artificial tears at varying glucose concentrations. (d) Glucose concentration in tears is read by capturing images of the lenses on the eye and analyzing RGB values via smartphone.^157^ Copyright 2020, with permission from Elsevier. (e) Schematics of contact lenses for single ion measurement and complete electrolyte analysis in tears. (f) Schematic of SiHG showing hydrophilic and hydrophobic interpenetrating networks; amphipathic H-ISFs are localized at the silicone-water interface; schematic cross-section of nonsilicone hydrogel lens with homogeneous structure. (g) pH-dependent equilibrium between neutral and anionic forms of 6HQ-C18, with photos of 6HQ-C18-labeled Biofinity CL at different pH under UV light. (h) Chloride quenching of SPQ-3 in water: i Emission spectra; ii Time-dependent decays (λ_ex_ = 355 nm).^150^ Copyright 2017, with permission from Elsevier. (i) and (j) Schematics of yellow color generation by CNP-PEG-GOx and fabrication of a CNP-PEG-GOx-laden lens via photopolymerization. (k) i The interface of the glucose colorimetric detection app shows an options menu; ii The color images with RGB profiles, and calculated tear glucose concentration. (l) Relationship between lens color and complementary color at different glucose concentrations, with images of CNP-PEG-GOx-laden lenses worn by healthy or diabetic rabbits.^104^ Copyright 2021 American Chemical Society.

#### Covalent Immobilization of Small-Molecule Fluorophores

Covalent bonding secures small-molecule fluorophores directly to the polymer backbone, preventing probe loss and ensuring stable signal output. An innovative structured silicone hydrogel (SiHG) contact lens has been advanced for precise monitoring of individual ion concentrations critical to diagnosing DED.^150^ The architecture comprises interpenetrating hydrophilic and hydrophobic networks with amphipathic H-ISFs localized at the silicone–water interface, contrasted with the homogeneous cross-section of a nonsilicone hydrogel lens (Figure 7e–f).These H-ISFs are integrated within the SiHG matrix, enabling real-time, noninvasive detection of key electrolytes like hydroxonium and chloride ions directly in the tear fluid. The fluorophores are covalently bonded to hydrophobic chains, ensuring their retention and functional stability within the lens structure, even under aqueous conditions.^150^ By combining wavelength-ratiometric and lifetime-based fluorescence sensing, these lenses enable precise detection of tear composition changes critical for DED management, independent of external light conditions. This dual-sensing approach allows for the monitoring of pH-dependent transitions between neutral and anionic forms of 6HQ-C18, with fluorescence shifts visible under UV light. Additionally, chloride ion dynamics are assessed using SPQ-3, analyzing both emission spectra and time-dependent decay rates to evaluate quenching effects (Figure 7g,h).^150^

#### Enzyme–Nanoparticle Composite Embedding

Embedding enzyme–nanoparticle hybrids combines catalytic activity with nanoparticle signal enhancement in a single microdomain. Recent advancements explored the use of contact lenses for glucose detection, incorporating cerium oxide nanoparticles (CNPs) for their colorimetric response.^104^ These CNPs are covalently linked to glucose oxidase using a biocompatible poly­(ethylene glycol) linker, forming a complex that is then integrated into the (hydroxymethyl)-methacrylate (HMA) polymer network. The yellow-color generation mechanism and lens fabrication are shown schematically in Figure 7i–j. Upon exposure to glucose, this enzymatic reaction catalyzes the conversion of glucose to gluconic acid and hydrogen peroxide, the latter of which oxidizes Ce^3+^ to Ce^4+^, resulting in a visible color change from colorless to yellow in approximately 1 min. This sensor’s sensitivity exceeds 0.1 mM for glucose detection. To facilitate practical application, a smartphone-based image processing algorithm has been devised to quantify this color change with high accuracy, equating to conventional spectrophotometric methods. (Figure 7k). In vivo validation was conducted using a transient diabetic rabbit model to assess the feasibility of CNP-PEG-GOx-laden contact lenses for tear glucose monitoring. Following glucose injection, a progressive color change was observed in the lenses, correlating with rising blood glucose levels (Figure 7l).^104^ These findings confirm the potential of this enzyme-based contact lens sensor for noninvasive glucose monitoring in diabetes management.

### Multifunctional Sensors across Various Regions of the Contact Lens

Multifunctional sensors integrated into different regions of the contact lens enable simultaneous sensing and therapeutic functions by leveraging variations in material composition or structural properties. These advanced designs allow for the concurrent detection of multiple biomarkers, environmental stimuli, or physiological changes while also serving as drug delivery platforms. Such an approach enhances the versatility of contact lens biosensors, making them highly effective for real-time, noninvasive medical diagnostics.

Structurally colored contact-lens sensors begin with the self-assembly of monodisperse SiO_2_ nanoparticles into an ordered, antiopal template, which is then infiltrated with a UV-curable hydrogel precursor and selectively etched to reveal a three-dimensional photonic crystal lattice (Figure 8a,b).^51^ When placed on the eye, variations in intraocular pressure deform the curved antiopal structure, shifting its Bragg reflection peak across the visible spectrum (Figure 8c–e). Simultaneously, the lens surface is functionalized with peptide-modified gold nanobowls that act as SERS substrates, enabling the label-free detection of MMP-9 down to nanomolar concentrations in tear fluid. In vivo tests in rabbits over 8, 16, and 24 h show no signs of corneal irritation or damage compared to commercial lenses, confirming biocompatibility for continuous ocular monitoring (Figure 8f). Another power-free design leverages an anodic aluminum oxide (AAO) nanopore array embedded within a soft hydrogel lens to integrate three capabilities—mechanical, pharmaceutical, and biochemical—in one platform (Figure 8g–i).^165–168^ For biomarker sensing, Interleukin-12p70 antibodies immobilized in the nanopores bind antigen from tear fluid, inducing a refractive-index shift that is quantified by a compact spectrometer with pg mL^–1^ sensitivity. Concurrently, therapeutic agents loaded into the AAO pores are released steadily over 30 days directly into the tear film, providing sustained drug delivery without the need for external pumps. Ex vivo studies on cadaver pig eyes demonstrate accurate IOP measurements between 10 and 50 mmHg, attributable to curvature-dependent optical shifts of the AAO film. The unified fabrication process, based on a single photocuring step, simplifies manufacturing and ensures robust multifunctionality for POC ocular diagnostics.

**Figure 8:**
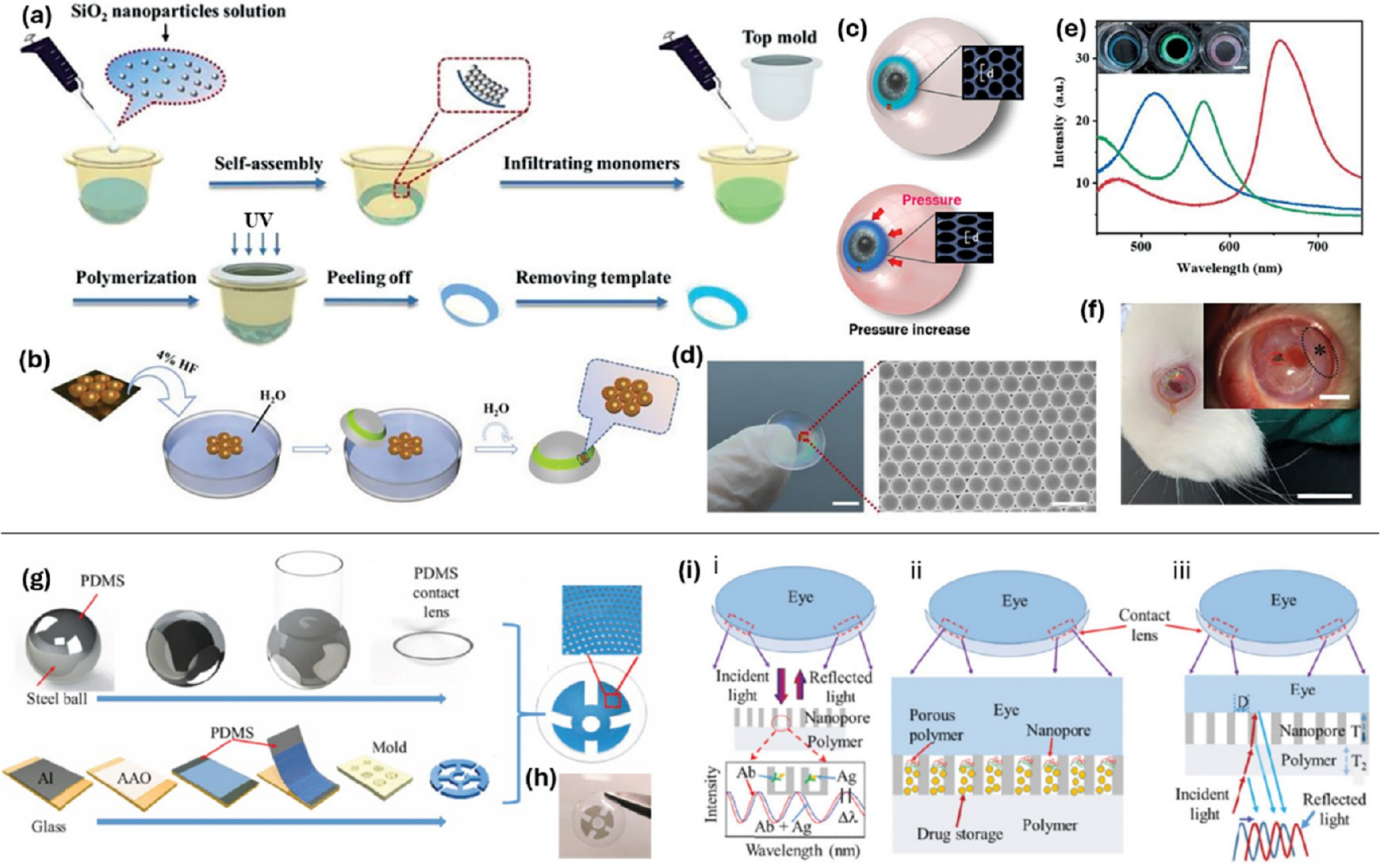
Multifunctional sensors across various regions of the contact lens. (a) The preparation process of structural color contact lenses involves nanoparticle self-assembly, monomer addition, polymerization, and template removal. (b) The process transfers AuNBs substrate from SiO_2_/Si wafer to the contact lens. (c) Schematic of the dual-functional contact lens sensor. (d) Photograph of AuNBs substrate on the structural color contact lens and SEM image of the substrate show scale bars of 5 mm and 1 μm, respectively. (e) Reflection spectra for contact lenses displaying blue, green, and red colors, with photographs inset (scale bar: 5 mm). (f) Photograph of a rabbit wearing a smart contact lens.^51^ © 2022 Ye, Y. Advanced Science published by Wiley-VCH GmbH. (g) Fabrication flow of the contact lens sensor includes patterning AAO nanopore thin film sensing modules. (h) Photographs of a fabricated smart contact lens device highlight the AAO thin film’s transparency in the pupil area compared to surrounding areas. (i) i Detailed view of the biomarker detection sensor where antibody–antigen binding shifts optical signal peaks. ii Details of the drug storage and release system using nanopores as containers with a porous silicone diffusion barrier for controlled release. iii Close-up of the IOP sensor showing peak shifts in the reflected optical signal corresponding to IOP changes.^165^ Copyright 2019 IEEE.

## Conclusions and Perspectives

Tear fluid-based biosensing has emerged as a promising noninvasive diagnostic approach, attracting significant attention in scientific, technological, and clinical fields.^25,26^ Optical contact lens biosensors, utilizing fluorescence, colorimetry, photonics, and plasmonic sensing, have rapidly evolved, providing a highly sensitive, cost-effective platform for early disease detection. These technologies hold immense potential in monitoring ocular diseases, and systemic conditions such as diabetes, cancers, sclerosis, and neurological disorders. However, key challenges remain in improving sensor sensitivity, reproducibility, and long-term stability for clinical applications. This review explores advancements in sensing techniques and fabrication methods, focusing on hydrogels and silicone hydrogels as primary substrate materials. Their high optical transparency, oxygen permeability, and biocompatibility make them well-suited for biosensing applications.^52,82^ The integration of sensors into contact lenses is achieved through three main approaches: surface fabrication, microstructure embedding, and molecular modification. Surface fabrication, employing coating, grafting, or direct ablation, enables straightforward sensor attachment and rapid analyte detection but is susceptible to mechanical wear. Embedding microstructures within the lens enhances sensor protection and allows multiplexed detection but may compromise comfort and optical performance. Molecular modification integrates sensing elements within the lens matrix, improving stability and sensitivity while maintaining transparency and flexibility. Contact lens sensors are advancing toward theranostic applications, integrating biosensing and drug delivery despite challenges in fabrication and material optimization.^165,169,170^ These advancements are expected to drive the clinical adoption of smart contact lenses, transforming wearable ocular diagnostics and treatment strategies.

### Challenges and Future Perspectives

Building on recent advances in optical contact lens biosensors, key challenges and future opportunities for clinical translation must be considered from the perspectives of sensing materials, fabrication methods, biosensor integration, and real-world deployment. (i) The correlation between tear biomarkers and systemic diseases remains an area of ongoing investigation. The relatively low concentration of certain analytes in tear fluid, coupled with variations due to external factors such as hydration, diet, and circadian rhythms, presents challenges in ensuring accuracy and reliability. Large-scale clinical studies are essential to validate tear biomarkers for diagnostic applications and to establish standardized reference ranges for disease monitoring. The specificity of biosensors must also be improved to minimize interference from structurally similar molecules in tear fluid, such as lactate in glucose detection.^171,172^ (ii) The commercial viability of smart contact-lens biosensors hinges on translating lab-scale processes—molecular imprinting, nanostructured coatings, and hydrogel polymerization—into GMP/ISO 13485–compliant, high-throughput manufacturing without sacrificing sensor precision, optical clarity, or mechanical resilience over days-to-weeks of wear. Robust surface-treatment, sterilization protocols, and strict batch-to-batch consistency are essential to ensure durable performance and wearer comfort. (iii) Clinical translation and adoption will require prospective human studies that establish diagnostic accuracy, long-term ocular biocompatibility within the ISO 10993 framework, and robust performance under tear-film dynamics and real-world conditions; designs should also minimize lens-replacement frequency.^173,174^ Signals to date are mixed: the SENSIMED Triggerfish soft lens for 24 h IOP-pattern monitoring received FDA De Novo classification in 2016 (DEN140017; Class II, 21 CFR 886.1925), establishing a regulatory precedent (with special controls) for wearable ocular sensors. By contrast, the Verily/Alcon glucose-sensing lens was discontinued in 2018 after studies showed variable/lagged tear–blood correlations and susceptibility to tear-film and environmental confounders. In 2023, Mojo Vision pivoted from developing smart lenses to commercializing micro-LED displays, underscoring the engineering, power, and safety hurdles associated with continuous on-eye electronics.^175–177^ Collectively, these cases indicate that while a pathway exists, significant validation and integration challenges remain. Going forward, developers should operate under an ISO 13485-compliant quality-management system and select the appropriate U.S. pathway—510­(k), De Novo, or PMA—based on intended use and risk; in the EU, conformity with MDR 2017/745 entails notified-body oversight, UDI assignment, and EUDAMED registration.^173,174,178,179^ Reimbursement planning, user compliance (comfort and handling), and end-to-end data governance aligned to HIPAA and GDPR should be integrated from the outset through coordinated industry–clinic–academia partnerships.^180,181^ (iv) The future of smart contact lens biosensors lies in their seamless integration with wireless, self-powered, and miniaturized point-of-care (POC) devices. High-resolution smartphone imaging for real-time optical readouts, combined with cloud-based data storage and AI-driven biomarker analysis, can enhance diagnostic accuracy and enable remote patient monitoring. Emerging AI algorithms can process complex fluctuations in biomarkers, providing predictive insights for personalized medicine. Furthermore, energy-efficient power solutions, such as biofuel cells or wireless energy transfer, could enable long-term operation without external charging, improving user convenience.

With ongoing interdisciplinary efforts in materials science, bioengineering, and digital healthcare, smart contact lens biosensors hold immense potential to revolutionize patient-centric diagnostics. By overcoming current challenges and advancing toward clinical implementation, these wearable devices may pave the way for noninvasive, real-time disease monitoring, transforming the future of ophthalmology and systemic disease management.
